# Defining Histological Patterns in Inherited Ichthyoses: Toward a Diagnostic Algorithm Based on 66 Confirmed Cases

**DOI:** 10.3390/dermatopathology13010009

**Published:** 2026-02-28

**Authors:** Kira Süßmuth, Vinzenz Oji, Jacqueline Bodes, Isabelle Jochum, Florian Muhs, Katalin Komlosi, Ingrid Hausser, Matthias Schmuth, Heiko Traupe, Judith Fischer, Dieter Metze

**Affiliations:** 1 Department of Dermatology and Allergology, Helios Klinikum Berlin-Buch, Campus of Medical School Berlin, 13125 Berlin, Germany; 2 Department of Dermatology, University Hospital of Münster (European Reference Network for Rare Skin Diseases (ERN Skin)), 48149 Münster, Germanymetzed@uni-muenster.de (D.M.); 3 Institute of Human Genetics, Medical Center—University of Freiburg, Faculty of Medicine, University of Freiburg (European Reference Network for Rare Skin Diseases (ERN Skin)), 79106 Freiburg, Germany; katalin.komlosi@uniklinik-freiburg.de (K.K.);; 4 Institute of Pathology, Heidelberg University Hospital, 69120 Heidelberg, Germany; 5 Department of Dermatology, Venerology an Allergy, Medical University Innsbruck, Karl Landsteiner Institute for Pediatric Dermatology and Rare Diseases (European Reference Network for Rare Skin Diseases (ERN Skin)), 6020 Innsbruck, Austria

**Keywords:** cornification disorders, ichthyoses, epidermis, dermatopathology, histological patterns, diagnostic algorithms

## Abstract

Background: Inherited ichthyoses are a heterogeneous group of disorders of cornification caused by mutations in genes encoding epidermal proteins. Clinically, patients with ichthyosis present with erythema, scaling, and occasionally blistering; some subtypes are syndromic. Accurate and timely diagnosis is essential for appropriate management and genetic counseling. Objectives: Diagnosis of ichthyosis typically relies on a combination of clinical features, histopathological and ultrastructural findings, immunohistochemistry, and molecular genetic testing. Dermatopathology can be particularly valuable in three diagnostic scenarios: (i) when the clinical diagnosis of ichthyosis is evident, but the specific subtype remains unclear; (ii) when differential diagnoses such as inflammatory dermatoses need to be excluded; and (iii) when molecular testing is unavailable or yields variants of uncertain significance. However, definitive classification according to current nomenclature requires molecular confirmation. Methods: Despite being a routine diagnostic tool in dermatology, histopathological criteria for ichthyoses remain ill-defined and diagnostically challenging. In this retrospective study, we systematically assessed histological features in 66 patients with confirmed ichthyosis. Results: Our analysis revealed six distinct histological patterns. Based on these, we propose a pattern-based diagnostic algorithm to support the histological classification of ichthyosis subtypes. Limitations: Although some rare subtypes were underrepresented, this cohort represents the largest and most heterogeneous group of molecularly confirmed ichthyosis cases analyzed histologically to date. Conclusions: Our findings highlight the diagnostic value of skin biopsies in inherited ichthyoses. The delineation of characteristic histological patterns and the development of a diagnostic algorithm may facilitate more accurate subtype identification, particularly in settings where genetic testing is limited or inconclusive.

## 1. Introduction

Ichthyoses are a heterogeneous group of inherited cornification disorders (Mendelian Disorders of Cornification, MeDOCs; new nomenclature: non-syndromic epidermal differentiation disorders (nEDDs) and syndromic epidermal differentiation disorders (sEDDs)) caused by gene variants affecting epidermal barrier function [1,2]. Patients with ichthyosis present with scaling and dry skin but may also have erythroderma and blistering. Some types of ichthyoses have syndromic manifestations (e.g., immunodeficiency, deafness, skeletal anomalies, neurological symptoms) and some are potentially life-threatening. The prevalence ranges from 1:200 to less than 1:1,000,000 [3,4,5,6].

Nowadays, the diagnosis of ichthyoses is based on clinical and histological findings and can be supported by electron microscopy and immunohistochemistry. For most dermatologists, clinical assignment to a subtype is very difficult or even impossible. However, the most reliable way of confirming ichthyosis is through molecular genetic analysis.

Histology is a fast, cost-effective and well-established method of dermatodiagnosis (although patients and physicians are often reluctant to perform a (minimally) invasive biopsy). In clinical practice, there are three scenarios in the diagnosis of ichthyosis where histology may be involved: (i) ichthyosis can be diagnosed with clinical certainty, but the subtype remains obscure; (ii) it is not clear whether the patient suffers from ichthyosis or an inflammatory dermatosis—a typical situation, for instance, in erythroderma in infants; (iii) molecular genetic diagnostics are not possible or detect variants with unclear significance.

To date, only a limited number of characteristic histological and ultrastructural findings in cornification disorders have been well documented in the literature [7,8,9,10,11]. Epidermolytic hyperkeratosis is the only well-defined pattern and is associated with keratin defects due to pathogenic variants in *KRT1*, *KRT2* and *KRT10*. Ross et al. characterized the histological features of keratinopathic ichthyosis and nevi in terms of epidermal changes and correlated these findings with the phenotype. Histological distinction between *KRT2* and *KRT1* or *KRT10* mutations was not always possible [7].

As previously shown, dermatopathology can be useful in the differential diagnosis of neonatal and infantile erythroderma [12,13]. In a cohort of 51 patients with erythodermic ichthyosis, histological analysis was useful in half of the patients, excluding those with Netherton syndrome [12].

To date, there is no valid definition of patterns in ichthyosiform skin disorders that allows for an algorithmic analysis of inflammatory skin diseases as described by Ackerman et al. [14]. Systematic analyses of larger cohorts are rare, and many publications are monographs, case reports or publications without genetic confirmation of these rare diagnoses [8,9,10,11].

Working in the Reference Center for Ichthyoses and Palmoplantar Keratoderma (ReCIP) in Münster collaborating with the Center for Cornification Disorders in Freiburg, we see many patients with hereditary cornification disorders in our consultations and carry out all the necessary analyses to obtain a diagnosis. With years of clinical experience, molecular genetic analysis, modern immunohistological staining methods and enzyme activity measurements, we could establish exact diagnoses in the majority of our patients, especially in recent years.

We systematically analyzed 87 biopsies from 66 patients with inherited ichthyosis. We then correlated the histological findings with clinical and genetic data, and we were able to define six histological patterns. On this basis, we developed a diagnostic algorithm that enables the histological classification of patients into specific ichthyosis subgroups and helps narrow down differential diagnoses.

## 2. Materials and Methods

### 2.1. Materials

In total, we identified 87 biopsies from 66 patients in whom the diagnosis was confirmed by clear clinical features and various diagnostic techniques. Genetic analysis was performed in 89.4% of patients.

It is noteworthy that the clinical evaluation, particularly the systematic screening for phenotypic findings, was performed by dermatologists from our centers with many years of experience in ichthyosis and keratinization disorders. All patients were phenotyped using a standardized questionnaire. As a member of the European Reference Network (ERN-Skin, Ichthyosis and Palmoplantar Keratoderma (IPK) subgroup), we have been involved in drafting the current version of a standardized questionnaire for ichthyosis and palmoplantar keratoderma. In addition, all patients have been discussed within our regular case conferences across specialized centers. Regarding clinical manifestations, we examined the presence and severity of ichthyosis (mild to moderate), palmoplantar hyperlinearity, palmoplantar keratoderma, erythroderma, presence of a collodion membrane at birth, (skin) malignancies, allergies, atopic dermatitis/diathesis and other dermatoses. In cases without records of disease severity, we collected data on the type of scaling. This study adhered to the Declaration of Helsinki and was approved by the local ethics committees (XTrau1, AN 2016-0260, 368/4.22, 454/AM1).

### 2.2. Histological Analysis

Histological analyses were performed by one of the authors, who is an experienced dermatopathologist (DM). For histology, 4 mm punch biopsies were taken, fixed in 4% formaldehyde, embedded in paraffin, sectioned and stained with hematoxylin and eosin [9,10].

The slides were evaluated semi-quantitatively using a scoring system (regarding the Stratum granulosum—absent: −−; reduced: −; normal: +; prominent: ++—and inflammation—no inflammation: −; mild: +; moderate: ++; severe: +++). In addition, the mean thickness of the stratum corneum (SC) hyperkeratosis and the vital epidermis was measured quantitatively (no hyperkeratosis: −; mild hyperkeratosis ≤ 0.1 mm: +; moderate hyperkeratosis > 0.1–0.2 mm: ++; severe hyperkeratosis > 0.2 mm: ++; no acanthosis ≤ 0.1 mm: −; acanthosis > 0.1–0.2 mm: +; acanthosis > 0.2–0.3 mm: ++; acanthosis > 0.3 mm: +++).

Psoriasiform hyperplasia was defined as regular hyperplasia with or without suprapapillary thinning. The presence of suprapapillary thinning was included under “other findings” in Table 1. For hyperplasia less than 0.1 mm, we did not differentiate between irregular and regular hyperplasia, because the assessment of suprapapillary epidermis seemed ambiguous. We performed a thorough histological evaluation of follicular and acrosyringeal hyperkeratosis. We also examined all biopsies for dilated capillaries in the papillary body and analyzed whether they were in close contact with the basal membrane (“kissing phenomenon”). Abnormal blood vessel findings were listed under other findings.

### 2.3. Genetic Analysis

In 59 patients, the clinical diagnosis was confirmed by genetic analysis. In all patients, genomic DNA was isolated from peripheral blood lymphocytes, followed by either Sanger sequencing or NGS methods (e.g., gene panel sequencing or whole-exome sequencing) [15].

Further diagnostics were performed via immunohistochemistry (n = 3) and in situ monitoring of transglutaminase 1 activity (n = 4) (Table 1).

Statistical analysis was carried out with IBM SPSS Statistics Version 28.1.1 (2021, IBM).

### 2.4. Cohort Demographics and Diagnostic Spectrum

We studied a total of 87 biopsies in 66 patients altogether, including 32 female and 34 male patients with the following diagnoses: ichthyosis vulgaris (new nomenclature for non-syndromic epidermal differentiation disorders (nEDDs) and syndromic epidermal differentiation disorders (sEDDs)) (*FLG*-nEDD) (n = 8), X-linked ichthyosis (*STS*-sEDD) (n = 10), autosomal recessive congenital ichthyosis (*TGM1*-nEDD; *SDR9C7*-nEDD; *ALOXE3*-nEDD; *ALOX12B*-nEDD; *NIPAL4*-nEDD; *ABCA12*-nEDD) (n = 19), autosomal dominant lamellar ichthyosis (*ASPRV1*-nEDD) (n = 2), epidermolytic ichthyosis (*KRT1*-nEDD; *KRT2*-nEDD; *KRT10*-nEDD) (n = 5), Netherton syndrome (*SPINK5*-sEDD) (n = 4), peeling skin disease (*CDSN*-nEDD) (n = 3), and rare (syndromic) subtypes (n = 15). Rare syndromic subtypes included keratitis–ichthyosis–deafness (KID) syndrome (*GJB2*-sEDD-KID); congenital hemidysplasia with ichthyosiform nevus and limb defects (CHILD) syndrome (*NSDHL*-sEDD-CHILD); ichthyosis follicularis, alopecia and photophobia (IFAP) syndrome (*MBTPS2*-sEDD-IFAP); severe dermatitis, multiple allergies and metabolic wasting (SAM) syndrome (*DSG1*-sEDD); MALT1 deficiency; autosomal recessive keratitis–ichthyosis–deafness (KIDAR) syndrome (*AP1B1*-sEDD-KID); and neutral lipid storage disease with ichthyosis and Sjögren–Larsson syndrome (*ALDH3A2*-sEDD). The median age at biopsy was 17 years (minimum: 3 days; maximum: 68 years). We had information on the biopsy site in 55 cases. All biopsies were taken from affected skin, with the majority from the extremities (Table 1).

Detailed information regarding whether biopsies from the extremities were obtained from ventral or dorsal sites was not available. However, when biopsies were taken from anatomical locations with characteristically thicker skin, such as over joints, knees, elbows or from the palms or soles, this information was documented and explicitly reported.

**Table 1:** Genetic, clinical and histological characteristics of patients with inherited ichthyosis.

Patient ID	Sex	Age	Biopsy Site	Diagnosis (Gene); (Mutation); (Diagnostic Method)	Cutaneous and Extracutaneous Symptoms	Histology							
						SC	SG	Acanthosis	Inflammation	FHK	AHK	Other Findings	Pattern(s)
1	f	9	upper limb	IV (*FLG1*) (2282del4 (Dra III)) (restriction enzyme digestion)	mild ichthyosis, palmoplantar hyperlinearity, keratosis pilaris, dyshidrosis lamellosa sicca (fingertip), atopic dermatitis	+, OK, lamellar	−−	+	+	FHK	n.a.		I
2	m	11	upper limb	IV (*FLG1*) (R501X (Nla III)) (restriction enzyme digestion)	moderate ichthyosis, severe palmoplantar hyperlinearity	+, OK, lamellar	−−	+	focal +	FHK	AHK		I
3	m	71	forehead	IV (*FLG1*) (2282del4 (Dra III)) (restriction enzyme digestion)	ichthyosis, pruritus, comorbidities: basalioma and SCC, lichen planopilaris	+, OK, lamellar	−	−	focal +	FHK	AHK	biopsy captured areas not affected by lichen planopilaris	I
4	m	1 month	lower limb	IV (*FLG1*) (R501X (Nla III)) (restriction enzyme digestion)	mild-to-moderate ichthyosis	+, OK, compact/lamellar	−−/−	−	−	n.a.	n.a.	n.a.	I
5	f	50	lower limb	IV (*FLG1*) (2282del4 (Dra III)) (restriction enzyme digestion)	ichthyosis, lymphadenopathy	+, OK, focal PK, compact/lamellar	−/+	+	+	n.a.	n.a.		I
6	m	12	1: right hand; 2: upper limb	IV (*FLG2*) (R501X (Nla III); 2282del4 (Dra III)) (restriction enzyme digestion)	ichthyosis, mild PPK, keratosis pilaris, atopic dermatitis (lower extremities)	1: +/++, OK, focal PK, lamellar; 2: ++, OK, focal PK, compact/lamellar	1; 2: −−/−	1; 2: +	1; 2: focal + (follicular)	1; 2: FHK	1: AHK	2: focal spongiosis; serum crust	I
7	m	60	lower limb	IV (*FLG2*) (R501X (Nla III); R501X (Nla III))) (restriction enzyme digestion)	ichthyosis, mild PPK	++, OK, com-pact/lamellar	−−	+	+/++	n.a.	AHK		I
8	f	29	upper limb	IV (*FLG2*) (restriction enzyme digestion)	moderate ichthyosis, atopic diathesis with recurrent eczema, hypohidrosis	+, OK, lamellar	−−/−	+	none	FHK	n.a.	BV: subepidermal dilatated	I
9	m	7	lower limb	XLI (*STS*) (STS activity test; *FLG*: restriction enzyme digestion (5 most common mutations) + panel/full *FLG* sequencing → no additional *FLG* mutation)	mild-to-moderate ichthyosis, atopic dermatitis	+, OK, lamellar	−	+	none	n.a.	AHK	focal spongiosis	I
10	m	17	1,2: lower limb	XLI (*STS*) (lipoprotein electrophoresis)	n.a.	1,2: +, OK, lamellar	1,2: +, focal ++	1: +/++, regular 2: −/+	1,2: +	n.a.	AHK with SG+		II
11	m	27	upper limb	XLI (*STS*) (STS activity test; *FLG*: restriction enzyme digestion (5 most common mutations) → no additional *FLG* mutation)	mild-to-moderate ichthyosis, no atopic dermatitis	+, OK, compact	+	+	none	n.a.	n.a.		II
12	m	25	posterior trunk	XLI (*STS*); (*STS*: Del Ex1–10); (NGS)	plaque-like scaling, light brown to dark brown, popliteal fossae with mild scaling, hypohidrosis	+, OK, lamellar	−	+/++, regular	none	n.a.	n.a.		I
13	m	40	posterior trunk	XLI (*STS*); (*STS*: Del Ex1–10); (NGS)	n.a.	++, OK, compact	+	+	none	n.a.	AHK with SG+		II
14	m	9	posterior trunk	XLI (*STS*); (Del *STS* Ex1–10; FLG, Exon 3: c.1501C>T p.Arg501* Class 5 Smith, 2006; ALOXE3, Exon 16: c.2008C>T p.Arg670Trp Class 3) (NGS)	n.a.	+, OK, lamellar	−	+	+	n.a.	AHK with SG +		I
15	m	58	upper limb	XLI (*STS* + *FLG1*); (*STS*: Del Ex1–10) (NGS)	white fine-to-lamellar scaling, atopic diathesis; astigmatismus	+, OK, compact/lamellar	+	+	+	n.a.	n.a.		II
16	m	51	lateral trunk	XLI (*STS* + *FLG1*); (Del *STS* Ex1–10; *FLG*, Exon 3: c.2282_2285del p.Ser761Cysfs*36 Class 5 Smith, 2005; *SPINK5*, Exon 9: c.715dup p.Cys239Leufs*6 Class 5 Raghunath, 2004); (NGS)	mild-to-moderate ichthyosis, keratosis pilaris upper and lower limbs, mild palmoplantar hyperlinearity	+, OK, lamellar	−−/−	+	+	n.a.	n.a.		I
17	m	46	anterior trunk	XLI (*STS* + *FLG1*) (NGS)	severe ichthyosis, no atopy	+, OK, compact	−−/−	+	+	n.a.	n.a.		I
18	m	7	upper limb	XLI (*STS* + *FLG2*) (R501X (Nla III); 2282del4 (Dra III)) (FISH; STS activity test; LC-MS/MS; restriction enzyme analysis (5 most common mutations))	severe fine-to-mild lamellar ichthyosis, atopic diathesis, palmoplantar hyperlinearity	+/++, OK, com-pact/lamellar	−−/−	++	+	n.a.	n.a.		I
19	m	50	trunk	ARCI (*ALOXE3*) (*ALOXE3*, Exon 15:c.1889C>T p.Pro630Leu Class 5 Eckl, 2005); (NGS)	CIE, mild phenotype; palmoplantar hyperlinearity; comorbidities: SCC, lichen planopilaris	++, OK, basket-weave-like/lamellar	−/+	++, regular	+	n.a.	AHK		II
20	f	3 days	upper limb	ACRI (*ALOXE3*) (external report)	mild generalized, white scaling	+/++, OK, basket-weave-like/compact/lamellar	+/++	++, regular	+	n.a.	n.a.		II
21	f	29	forehead	ARCI (*ALOXE3*) (external report)	extreme hypohidrosis, fine lamellar scaling, kinking ear, many papillomatous nevi	+, OK, lamellar	+	+/++	+	n.a.	n.a.	BV: dilatated, kissing vessels	II
22	f	47	upper limb	ARCI (*ALOX12B*); (*ALOX12B*, Exon 12:c.1533+1G>T p.spl? Class 5; *ALOX12B*, Exon 15:c.2005_2037dup p.Leu669_Arg679dup Class 3); (NGS)	lichenification (hyperkeratotic), fine-to-mild lamellar scaling, face included, mild PPK with mild hyperlinearity	+, OK, compact/lamellar	+/++	++, regular	+	n.a.	AHK	BV: dilatated	II
23	f	25	1: posterior trunk; 2: anterior trunk	ARCI (*ALOX12B*); (*ALOX12B*, Exon 15:c.2041A>T p.Lys681* Class 5); (NGS)	CIE, very high IgE levels, milk allergy	1: +, OK/PK, lamellar; 2: ++, OK, compact	1: +/++; 2: ++	1: ++; regular, no neutrophils 2: ++, regular	1: ++; 2: +	n.a.	n.a.	1; 2: BV: dilated; focal serum crust; spongiosis; 1: PK with roundish nuclei and in a broad front; SG focally very prominent	II
24	f	3	lower limb	ARCI (*SDR9C7*); (*SDR9C7*, Exon 2: c.346G>A p.Gly116Ser Klasse 4; SDR9C7, Exon 2: c.551A>G p.Asp184Gly Class 5); (NGS)	lamellar ichthyosis	+, OK, compact/lamellar	+/++	++, regular	none	n.a.	AHK	BV: dilatated	II
25	f	13	gluteal	ARCI (*SDR9C7*); (*SDR9C7*, Exon 2: c.415G>A p.Glu139Lys Class 3–4; *SDR9C7*, Exon 2: c.551A>G p.Asp184Gly Class 5; *ABCA12*, Exon 11: c.1287+9T>C p.spl? Class 3); (NGS)	CIE, mild lamellar dark scaling	+, OK, lamellar	+	++, regular	+	FHK	AHK		II
26	f	11 months	lower limb	ARCI (*NIPAL4*) (*NIPAL4*, Exon 4: c.527C>A p.Ala176Asp Class 5 Lefevre, 2005); (NGS)	lamellar ichthyosis, focal yellowish PPK	++/+++, OK, lamellar	++	++, regular	+/++	FHK	AHK	BV: dilated; SC thicker than epidermis	II
27	f	30	posterior trunk	ARCI (*NIPAL4*) (*NIPAL4*, Exon 4: c.527C>A p.Ala176Asp Class 5 Lefevre, 2005); (NGS)	lamellar ichthyosis; comorbidity: actinic keratosis	+, OK, lamellar/compact	+	+/++, regular	+	FHK	n.a.		II
28	f	50	shoulder	ARCI (*NIPAL4*); (*NIPAL4*, Exon 2: c.463+5G>A p.spl? Class 5; *NIPAL4*, Exon 4:c.527C>A p.Ala176Asp Class 5 Lefevre, 2005); (NGS)	CIE	++, OK, focal PK, com-pact/lamellar	++	++, regular	+	n.a.	n.a.	BV: dilated; focal spongiosis	II
29	m	43	upper limb	ARCI (*NIPAL4*); (NIPAL4, Exon 2: c.463+5G>A p.spl? Class 5); (NGS)	lamellar ichthyosis, collodion baby, hypohidrosis	+/++, OK, lamellar/basket-weave-like	+	+/++, regular	+	n.a.	n.a.	BV: dilated	II
30	m	13	upper limb	ARCI (*TGM1*) c.60delT (p.Thr21Profs*90); c.60delT (p.Thr21Profs*90) (NGS)	lamellar ichthyosis with gray and bright-brown scaling, PPK	++, OK, compact > lamellar	+	++, regular	+	n.a.	n.a.		II
31	m	56	upper limb	ARCI (*TGM1*); *TGM1*, Exon 6: c.877-2A>G p.spl? Class 5 Huber, 1995; *TGM1*, Exon 8: c.1166G>C p.Arg389Pro Class 5 Shevchenko, 2000); (NGS; TGM1 Assay: reduced)	lamellar ichthyosis	++, OK, compact/lamellar	+	+/++, regular	+	FHK	n.a.	BV: dilated	II
32	f	n.k.	n.k.	ARCI, bathing suit ichthyosis (*TGM1*); (*TGM1*, Exon 5: c.845A>G p.Gln282Arg Class 3–4; *TGM1*, Exon 5: c.845A>G p.Gln282Arg Klasse 3–4); (TG1 Assay: reduced)	extreme scaling of abdomen (cranial of umbilicus), yellowish PPK; growth retardation;	++, OK, compact/lamellar	+	+/++, regular	+	FHK	AHK	BV: dilated	II
33	f	12	gluteal	ARCI (*TGM1*); (*TGM1*, Exon 10: c.1466A>G p.Tyr489Cys Class 3; *TGM1*, Exon 10: c.1472C>T p.Thr491Met Class 5 Rodríguez-Pazos, 2011); (NGS)	lamellar ichthyosis	+++, OK, focal PK, compact/lamellar	++	++, regular	+	FHK	n.a.	BV: dilated, kissing vessels	II
34	m	2 months	upper limb	ARCI (*TGM1*); (c.788G>A, p.(Trp263*; c.1166G>A, p.Arg389His); (NGS; TG1 Assay: focal absent)	lamellar ichthyosis, focal erythema	+, OK, lamellar/compact	+	+	+	FHK	n.a.		II
35	f	44	posterior trunk	ARCI (*SDR9C7* + *FLG1*); (*SDR9C7*, Exon 3: c.561-1G>T p.spl? Class 4; *SDR9C7*, Exon 3: c.561-1G>T p.spl? Class 4; FLG, Exon 3: c.2282_2285del p.Ser761Cysfs*36 Class 5 Smith, 2005); (NGS)	white mild lamellar scaling, mild erythroderma, tinea plantar	+, OK, lamellar/basket-weave-like	−/+	+/++, regular	+	n.a.	n.a.		II
36	m	15	upper limb	harlequin ichthyosis, overlap lamellar ichthyosis (*ABCA12*) (c.2140C>T(Arg714*); 2341T>A(Cys781Ser)); (*ABCA12*, Exon 17: c.2140C>T, p.(Arg714*) (Class 5); *ABCA12*, Exon 18: c.2341T>A, p.(Cys781Ser) (Class 4)); (NGS)	severe erythroderma and scaling; clinical overlap of lamellar ichthyosis with harlequin ichthyosis	+, OK, focal PK, compact	+	++/+++, regular	++	n.a.	normal	neutrophils in SC; BV: dilatated, kissing vessels	II
37	m	20	upper limb	harlequin ichthyosis (*ABCA12*) (c.3829+3A>G, Spleiß-Mutation, Intron 26); c6722_6723delGA (p.Arg224Ilefs*4)) (NGS)	severe erythroderma and scaling	+, OK, focal PK, compact	−−	++, regular	++	n.a.	n.a.	neutrophils in SC; BV: dilatated, kissing vessels, suprapapillary thinning, papillomatosis	I
38	f	n.k.	n.k.	ADLI (*ASPRV1*); (*ASPRV1*, Exon 1: c.940C>A, p.(Pro314Thr) Class 4; *CARD 14*, Exon 6: c.[925C>T], p.[Arg309Trp] Class 3); (NGS)	mild lamellar scaling, mild diffuse PPK	+/++; OK/PK, lamellar	+	+, irregular	+	n.a.	n.a.		III
39	f	n.k.	n.k.	ADLI (*ASPRV1*) (NGS)	mild lamellar scaling, mild diffuse PPK	+/++; OK/PK, lamellar	+	+, irregular	none	n.a.	n.a.		III
40	m	3 months	n.k.	EI (n.a.)	nevoid epidermolytic ichthyosis, neck and joints accentuated, face involved, no PPK; hypohidrosis	++/+++, OK, lamellar/basket-weave-like	+	+	+	n.a.	n.a.	EHK continuous, suprabasal	IV
41	m	39	trunk	EI (*KRT1*) (c.1434G>T (p.Glu478Asp)) (exome sequencing)	generalized mild scaling, PPK; comorbidity: urticaria	−, OK, basket weave	+	+	++/+++	n.a.	n.a.	EHK continuous, suprabasal	IV
42	m	15	upper limb	EI (*KRT1*); (*KRT1*, Exon 7: c.1457T>A p.Leu486Gln Mosaik 20% Class 5 Bygum, 2013); (NGS)	hyperkeratosis and erosions over joints, PPK	++, OK, basket-weave-like/lamellar/focal compact	+	++, regular	+	n.a.	n.a.	EHK almost continuous, suprabasal, papillomatosis	IV
43	m	68	knee	EI (*KRT10*) (external report)	ichthyotic scaling lesions with blistering and erosions, yellowish PPK	+++, OK, compact/lamellar	+	++, regular	+	n.a.	n.a.	EHK, continuous, suprabasal, suprapapillar epidermis thin;	IV
44	f	16	knee	EI (*KRT10*) (exome sequencing) (external report)	clinical similarities to SEI, brown hyperkeratosis and scaling	+++, OK, compact/lamellar	+	++, regular	+	n.a.	n.a.	EHK non-continuous, suprabasal, papillomatosis	IV
45	f	21	neck	NTS (*SPINK5*) (sequencing; IHC: LEKTI absent)	generalized ichthyosiform erythroderma, severe pruritus	+, OK, focal PK, lamellar	+/++	++, regular	++	n.a.	n.a.	BV: dilatated, kissing vessels, corneocytes detached (“flying birds”), suprapapillar epidermis thick	VI
46	f	7	gluteal	NTS (*SPINK5*) (IHC: LEKTI absent)	ichthyosiform erythroderma with aspects of ichthyosis linearis circumflexa; severe pruritus, vitamin d deficiency	1: +, OK, focal PK, basket-weave-like; neutrophils and lymphocytes; 2: +, OK, focal PK, basket-weave-like	1: −−/+; 2: −−/+	1: ++, regular; 2: ++, regular	1,2: ++ with eosinophils in 2	FHK	n.a.	BV: dilatated, kissing vessels, suprapapillar epidermis normal/thin, focal spongiosis, hyperplasia verruciform; ILVEN-like	VI
47	f	4 months	n.k.	NTS (*SPINK5*); (*SPINK5*, Exon 12: c.1092+1G>A p.spl? Class 5; ) *SPINK5*, Exon 16: c.1431-12G>A p.spl? Class 5 Raghunath, 2004); (NGS; IHC: *LEKTI* absent)	erythroderma, scaling, hypotrichosis, bacterial (*Staph. aureus*) and mycotic superinfections (*T. rubrum*)	1: +, OK, focal PK; 2: +, OK, focal PK	1: −−/+; 2: −−/+	1: ++, regular; 2: ++, regular	++	FHK	n.a.	1; 2: BV: dilatated, kissing vessels, suprapapillar epidermis thin, spongiosis; 2: intraepidermal neutrophils and lymphocytes and eosinophils	VI
48	m	1	gluteal	NTS (*SPINK5*) (c.1048C>T (p.R350*);c.1431-12G>A,IVS15-12G>A))	erythroderma with few scales	+, PK	−−	++, regular	++	n.a.	n.a.	BV: dilatated, kissing vessels, suprapapillar epidermis thin, neutrophils in SC	VI
49	m	18	upper limb	PSD (*CDSN*) (IHC: CDSN absent) (whole-genome linkage analysis via chip-based SNP analysis; sequencing)	severe atopic dermatitis (EASI 41.5), diffuse peeling and inflammation of the skin (esp. on extremities), severe pruritus	++, OK/PK, subcorneal clefting	−−/++	+++, regular	++	n.a.	n.a.	suprapapillar epidermis thin, clumsy rete ridges	VI
50	m	7	trunk	PSD (*CDSN*) (whole-genome linkage analysis via chip-based SNP analysis, sequencing)	mild phenotype, localized inflammation, multiple nevi, multiple allergies	1–3, 8–14: +, OK, basket-weave-like and lamellar; 4: +, OK, focal PK; 5: +, OK, focal (circumscribed) PK; 6: see histology 1, lamellar; 7: +, almost only PK; 8: SC missing; 14: focal (circumscribed) PK	1, 2, 4, 6, 9–14: ++, single acantholytic cells; 3: +++; 5: +/++; 7: +/++, partly −−; 8: SG +/++, partly −−	1, 6, 9, 10, 12, 13: +; 2, 3, 5, 7, 8, 11; 14: ++, regular	1; 2; 4–5; 9; 11–13: +; 3: ++; 7–10; 14: ++	n.a.	n.a.	1: suprapapillar epidermis thin, BV: dilated, kissing vessels, subcorneal clefting; 5: erythrocytes in cleft; 6: neutrophils in upper epidermis, spongiosis; 7: focal neutrophils	VI
51	f	9/13/16	trunk	PSD (*CDSN*) (whole-genome linkage analysis via chip-based SNP analysis, sequencing)	diffuse peeling and inflammation of the skin, severe pruritus, multiple nevi, verrucae vulgaris, vitamin D deficiency	1: ++, PK, focal OK 2: PK, focal OK, basket-weave-like 3: +, OK, basket-weave-like/lamellar	1: −−, 2: +/−− 3: ++	1: +++, irregular 2; 3: ++, irregular	1; 3: ++ 2: +	FHK	AHK	1: suprapapillar epidermis thin, BV: dilated, kissing vessels, neutrophils and bacteria in SC, only focal subcorneal clefting; 2: BV: dilated; 3: SG very prominent (3–4 layers), subcorneal clefting; 1, 2: acantholytic cells	VI
52	f	51	trunk.	mono-symptomatic CHILD (CHILD nevus) (*NSDHL*) (c.1046A>G (p.Y349C))	right body side with erythema and scaling	+++, OK, focal PK, compact	+, focal missing	+++, irregular	+/++	n.a.	n.a.	no neutrophils in SC, ILVEN-like histology, papillomatosis, BV: dilatated, no xanthoma cells	VI
53	f	n.k.	n.k.	CHILD	unilateral inflammation and scaling, scoliosis	++, OK, focal PK, compact	+, focal −−	++, irregular	++	n.a.	n.a.	neutrophils in SC, BV: dilatated, kissing vessels, clumsy rete ridges, few xanthoma cells	VI
54	m	n.k.	n.k.	IFAP syndrome (*MBTPS2*) (c.1427T>C (p.L4675))	hyperkeratosis on back, dry skin, no erythema, no collodion baby, no sweating	+, OK, lamellar/basket-weave-like	−	+	none	n.a.	n.a.	hair follicle rudimentary/atrophic, BV: dilatated, club-shaped rete ridges	I
55	f	33	upper limb	IFAP syndrome	n.a.	++/+++, OK, basket-weave-like/compact	+	−	none	FHK	n.a.		I
56	m	n.k.	n.k.	SAM syndrome (*DSP*) (c.1748C>T (p.L583P))	severe erythroderma, PPK, severe life-threatening infections, dilated cardiomyopathy	+, PK	−−	+++, regular	++	n.a.	n.a.	neutrophils in SC, BV: dilatated, no kissing vessels, club-shaped rete ridges, suprapapillary epidermis thin, widening of the intercellular spaces	VI
57	f	11 months	n.k.	MALT1 deficiency (*MALT1*) (external report)	dry and scaling skin, severe infections, brain atrophy, syndactyly	+, OK, focal PK	−−	++, regular	+++	−	−	neutrophils in SC, BV: dilatated, no kissing vessels, many lymphocytes, erythrocytes, neutrophils, eosinophils, long rete ridges, suprapapillar epidermis thin, spongiosis, inflammation in hair follicle, dyskeratosis in adnexa	VI
58	m	n.k.	n.k.	KID	massive hyperkeratosis, multiple SCC	+++, OK, lamellar	++	+	++	n.a.	n.a.	BV: dilatated, kissing vessels, papillomatosis	II
59	f	39	posterior trunk	KID	multiple hyperkeratoses	+++, OK, lamellar	−−/−	+	+	FHK	AHK	long and thin rete ridges, suprapapillar thinning	I
60	f	n.k.	n.k.	KID	n.a.	+++, OK, lamellar/basket-weave-like	−/+	+	++	FHK	n.a.	BV: dilatated, kissing vessels, papillomatosis, hyperplastic sweat glands	II
61	f	21	trunk	KID (*GJB2*); (*GJB2*, Exon 2: c.148G>A p.Asp50Asn Class 5 van Steensel, 2002); (NGS)	multiple follicular hyperkeratoses	+++, OK, lamellar	−/+	+	+	FHK	AHK		II
62	f	5	gluteal	SLS	fine scaling on trunk and extremities, cobblestone scaling in neck area, severe pruritus	++, OK, compact/basket-weave-like	++	+	+	FHK	n.a.	BV: normal steeple-shaped appearance	II
63	f	13	posterior trunk	SLS (*ALDH3A2*) (c.682C>T)	generalized lichenification and fine lamellar scaling	+, OK, basket-weave	−	+	+	n.a.	n.a.		I
64	m	22	upper limb	Chanarin–Dorfman syndrome (*ABHD5*) (c.838C>T)	generalized fine-to-midlamellar scaling	+, OK, focal PK, compact/lamellar	+	+	+	FHK	n.a.	discrete vacuolization of basal keratinozytes	II
65	f	14	lower limb	Conradi–Hünermann–Happle syndrome/X-linked dominant chondrodysplasia punctata (*EBP*)	brown lamellar scaling, follicular hyperkeratosis, scarring alopecia	1 (2 years old): +, OK; 2 (14 years old): ++, OK, compact/basket-weave-like	1: −−/−; 2: −	1: +; 2: +/++	1: +; 2: +	1: FHK; 2: n.a.	1: AHK; 2: n.a.	1: club-shaped and broadened rete ridges, von Kossa stain positive 2: BV slightly dilatated, clumsy rete ridges	I
66	m	3 months	trunk	KIDAR syndrome (mutation found in brother: *AP1B1* mutation)	n.a.	+, OK, compact/basket-weave-like	++	+	+	FHK	AHK	BV: dilatated, kissing vessels, hair follicle without pathological finding	II

Abbreviations: AHK, acosyringial hyperkeratosis; ADLI, autosomal dominant lamellar ichthyosis; ARCI, autosomal recessive congenital ichthyosis; BV, blood vessel; CIE, congenital ichthyosiform erythroderma; CDSN, corneodesmosin; EHK, epidermolytic hyperkeratosis; EI, epidermolytic ichthyosis; f, female; FHK, follicular hyperkeratosis; FLG (FLG1: one mutation; FLG2: two mutations), filaggrin; IFAP, ichthyosis follicularis, alopecia and photophobia; IHC, immunohistochemistry; ILVEN, inflammatory linear verrucous epidermal nevus; IV, ichthyosis vulgaris; KH, keratohyalin; KHG, keratohyalin granule; KID syndrome, keratitis ichthyosis deafness syndrome; KIDAR syndrome, autosomal recessive keratitis ichthyosis deafness syndrome; KRT, keratin; LI, lamellar ichthyosis; n.a., not assessable; m, male; n.k., not known; NGS, next-generation sequencing; NTS, Netherton syndrome; OK, orthokeratosis; PH, psoriasiform hyperplasia; PK, parakeratosis; PPK, palmoplantar keratoderma; PSD, peeling skin disease; SAM syndrome, severe dermatitis, multiple allergies and metabolic wasting syndrome; SCC, squamous cell carcinoma; SEI, superficial epidermolytic ichthyosis; SICI, self-improving congenital ichthyosis; SLS, Sjögren–Larsson syndrome; SNP, single-nucleotide polymorphism; STS, steroid sulfatase; XLI, X-linked ichthyosis. Classification of patterns—see Discussion and Table 2; ID numbers refer to patients in Table 1. Table 1 starts with common and non-syndromic ichthyoses and ends with rare and syndromic subtypes. If available, we added data on mutations. In patients with more than one available biopsy, we numbered the biopsies. SC (stratum corneum): no hyperkeratosis: −; mild hyperkeratosis ≤ 0.1 mm: +; moderate hyperkeratosis > 0.1–0.2mm: ++; severe hyperkeratosis > 0.2mm: +++; OK/PK: staggering. SG (stratum granulosum): absent: −−; reduced: −; normal: +; prominent: ++. Acanthosis: no hyperplasia ≤ 0.1 mm: −; mild hyperplasia > 0.1–0.2mm: +; moderate hyperplasia > 0.2–0.3mm: ++; severe hyperplasia > 0.3mm: +++. Inflammation: no inflammation: −; mild: +; moderate: ++; severe: +++. Psoriasiform hyperplasia is defined by regular hyperplasia with or without suprapapillary thinning. If suprapapillary thinning was found, we added it under “other findings [SK1.1]”.

## 3. Results

### 3.1. Histological Analysis of Different Subtypes of Ichthyosis

#### 3.1.1. Common Ichthyoses: Ichthyosis Vulgaris (IV) and X-Linked Ichthyosis (XLI)

**Figure 1:**
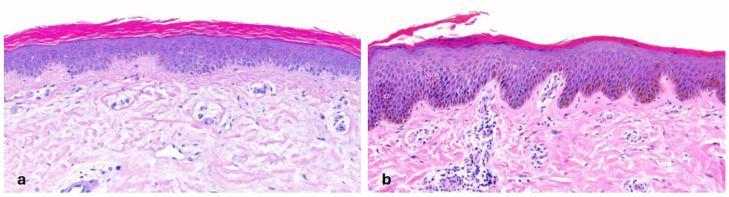
**Ichthyosis vulgaris (*FLG*-nEDD)**. (**a**): Orthohyperkeratosis, lamellar type, absence of stratum granulosum and mild acanthosis. Pattern 1 (ID 8, HE, original magnification ×40). (**b**): Orthohyperkeratosis, compact type, absence of stratum granulosum and acanthosis. Note focal parakeratosis and formation of a stratum granulosum and some spongiosis associated with an inflammatory infiltrate. Pattern 1 (ID 5, HE, original magnification ×40).

In IV, due to *filaggrin (FLG)* mutations, all histologies showed **orthohyperkeratosis with a reduced or absent stratum granulosum** (SG). SC was lamellar or compact. Mild acanthosis was observed in all patients except for two patients with only one *FLG* mutation. Notably, mild inflammation was common (75%), but only one patient had clinical erythema. A history of atopic dermatitis (AD) was reported in three patients, one of whom had histologically confirmed spongiosis and serum crust. Interestingly, prominent hyperkeratosis and absent SG were not significantly more common in patients with biallelic *FLG* mutations compared to those with one mutation (*p* = 0.464; *p* = 0.464). Follicular or acrosyringial hyperkeratosis was observed in six patients (Figure 1).

**Figure 2:**
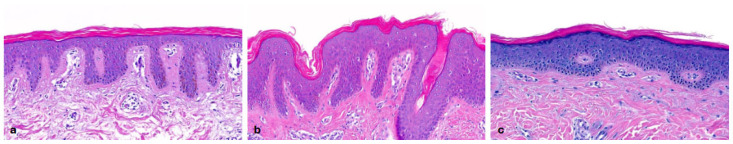
**X-linked ichthyosis (*STS*-sEDD)**. (**a**): Orthohyperkeratosis, lamellar type, with reduced stratum granulosum, and regular, psoriasiform acanthosis. Pattern 1 (ID,12, HE, original magnification ×40). (**b**): Orthohyperkeratosis with absence of stratum granulosum, regular acanthosis and mild inflammation. Note follicular hyperkeratosis. Pattern 1 (ID18, HE, original magnification ×40). (**c**): Orthohyperkeratosis and a well-developed stratum granulosum, mild acanthosis. Pattern 2 (ID 11, HE, original magnification ×40).

In the steroid sulfatase deficiency group causing XLI, 6 out of 10 patients showed orthohyperkeratosis with a reduced or absent SG; the rest had orthohyperkeratosis and a well-developed SG. Of note, three patients had additional *filaggrin* mutations with and without reduced SG. The differences in reduced or normal SG between the groups with and without additional *FLG* mutations, respectively, were not significant (*p* = 0.52). The SC was compact or lamellar. All patients showed at least mild acanthosis, and two patients showed moderate regular hyperplasia. Inflammation was present in six patients. Acrosyringeal hyperkeratosis was observed in four patients (three without additional *FLG* mutation), with the formation of an SG in the acrosyringium in three patients (Figure 2). Hair follicles could not be assessed.

#### 3.1.2. Autosomal Recessive Congenital Ichthyosis (ARCI), Including Harlequin Ichthyosis

**Figure 3:**
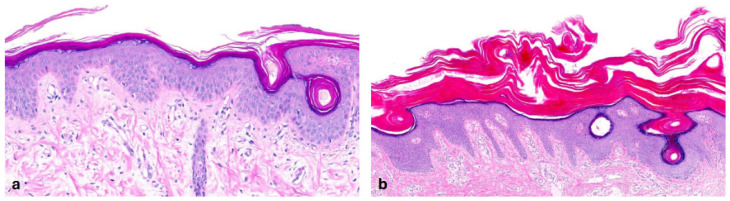
**Autosomal recessive congenital ichthyosis (ARCI)** (*TGM1*-nEDD). (**a**): Moderate orthohyperkeratosis and preserved stratum granulosum, regular acanthosis and follicular hyperkeratosis. Pattern 2 (ID32, HE, original magnification ×40). (**b**): Pronounced orthohyperkeratosis and well-developed stratum granulosum, regular acanthosis and follicular hyperkeratosis. Pattern 2 (ID33, HE, original magnification ×20).

**Figure 4:**
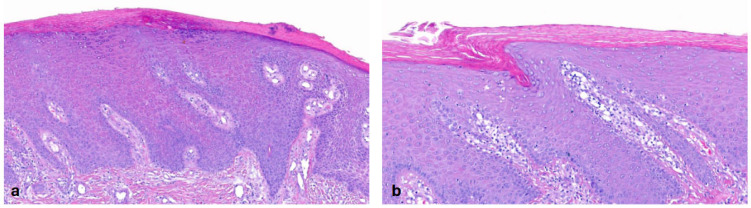
**Harlequin ichthyosis (*ABCA12*-nEDD)**. (**a**): Orthohyperkeratosis with a prominent stratum granulosum and regular acanthosis. Dilated vessels in the papillary dermis close to the epidermis. Pattern 2 (ID 36, HE, original magnification ×40). (**b**): Orthohyperkeratosis, absence of a stratum granulosum and prominent, regular acanthosis containing a few neutrophils. Dilated vessels in the papillary dermis close to the epidermis and thinning of the suprapapillary epidermis are psoriasis-like features. Pattern 1 (ID 37, HE, original magnification ×40).

In the heterogeneous subset of ARCI, we recruited 19 patients. Three of them had *ALOXE3* mutations, two had *ALOX12B* mutations, three had *SDR9C7* mutations and four had *NIPAL4* mutations in genetic testing [15]. We also included five patients with *TGM1* mutations and two patients with harlequin ichthyosis due to *ABCA12* mutations. Almost all patients showed **orthohyperkeratosis and a well-developed stratum granulosum** (Figure 3). Mild-to-moderate hyperkeratosis presented as lamellar to compact. One patient with transglutaminase 1 (TG1) deficiency showed severe orthohyperkeratosis and one patient with a *NIPAL4* mutation showed moderate-to-severe orthohyperkeratosis. Of note, one patient with an *SDR9C7* mutation had a reduced-to-normal SG, possibly due to an additional *FLG* mutation detected during routine genetic analysis. Mild-to-moderate epidermal acanthosis was observed in the majority of patients. Mild inflammation was common (n = 14), and moderate inflammation was rare (n = 2; *ALOX12B*, *NIPAL4*). The remaining patients had biopsies with different patterns of inflammation (mild to moderate, or one biopsy with moderate and one with mild-to-moderate inflammation). Follicular hyperkeratosis (FHK) and acrosyringial hyperkeratosis (AHK) were described in all patients, whenever hair and sweat glands could be assessed. In contrast to the other ARCI patients, the two patients with harlequin ichthyosis showed compact orthohyperkeratosis with focal parakeratosis, moderate-to-severe acanthosis, moderate inflammation and a stratum corneum with neutrophils. The SG was absent in the patient with isolated harlequin ichthyosis. In one patient with clinical overlap with lamellar ichthyosis (ID 36), the SG was regular (Figure 4).

#### 3.1.3. Autosomal Dominant Lamellar Ichthyosis

**Figure 5:**
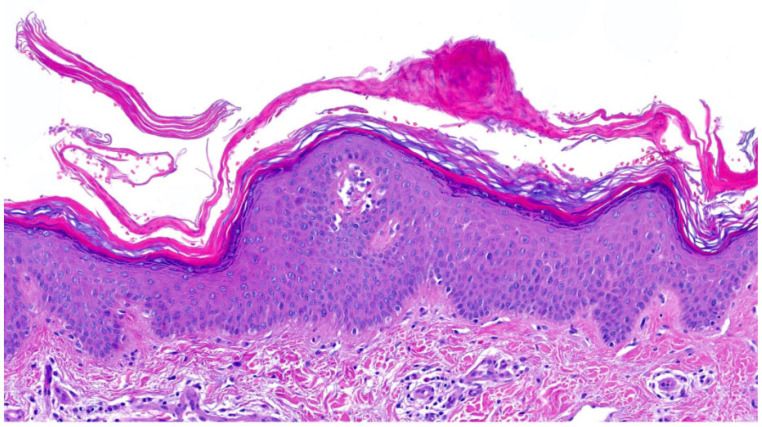
**Autosomal dominant lamellar ichthyosis (*ASPRV1*-nEDD)**. Hyperkeratosis with ortho- and parakeratosis and preserved stratum granulosum with irregular acanthosis. Pattern 3 (ID 38, HE, original magnification ×30).

Autosomal dominant lamellar ichthyosis was first described in 1984 by Traupe et al. [16] and genetically confirmed in 2020 by Boyden et al. [17], who reported a gene defect involved in filaggrin degradation. Both patients included in our study with a heterozygous mutation in the *ASPRV1* gene showed lamellar **hyperkeratosis with ortho- and parakeratosis with a preserved stratum granulosum**. The epidermis showed irregular acanthosis and one patient had mild inflammation (Figure 5). Clinically, these patients also presented with a diffuse PPK. Although the pattern of inheritance is different from ARCI, clinical differentiation between these two subtypes can be challenging.

#### 3.1.4. Keratinopathic Ichthyosis

**Figure 6:**
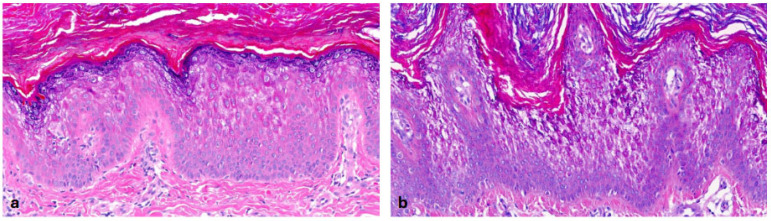
**Keratinopathic ichthyosis (*KRT10*-nEDD)**. (**a**): Pronounced orthohyperkeratosis, acanthosis, irregular keratohyalin granules and hypereosinophilic keratinocytes with vacuolated cytoplasma characteristic of epidermolytic hyperkeratosis. Pattern 4 (ID43, HE, original magnification ×40). (**b**): Pronounced orthohyperkeratosis, acanthosis, irregular keratohyalin granules and vacuolated keratinocytes with prominent hypereosinophilic granules (granular degeneration) characteristic of epidermolytic hyperkeratosis. Pattern 4 (ID44, HE, original magnification ×40).

In the group of keratinopathic ichthyoses, we included patients with mutations in *keratin (KRT)1* (n = 2) and *KRT10* (n = 2). One patient did not have a genetic report but had clear clinical and histological features of keratinopathic ichthyosis. All patients had orthohyperkeratosis. Acanthosis was mild to moderate and regular. All patients exhibited a pattern of suprabasal **epidermolytic hyperkeratosis** characterized by irregular keratohyalin granules and vacuolated keratinocytes with hypereosinophilic granules (granular degeneration) (Figure 6); it was non-discontinuous in one patient and continuous in all remaining cases. Hair follicles and acrosyringia were not assessable. Moderate-to-severe inflammation was observed only in one patient with a *KRT1* mutation who had a comorbidity of urticaria. The skin biopsy was taken from a non-urticaria area. Inflammation was mild in the other patients. One patient with a *KRT10* mutation presented PPK, which is typically seen in patients with a *KRT1* mutation.

#### 3.1.5. Netherton Syndrome (NTS)

**Figure 7:**
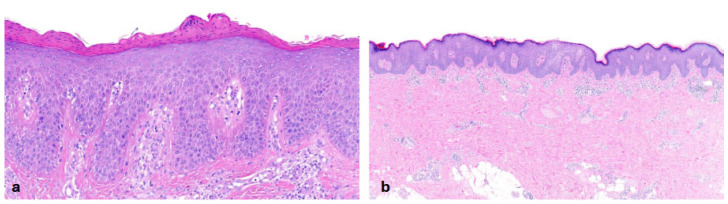
**Netherton syndrome (*SPINK5*-sEDD)**. (**a**): Psoriasis-like pattern with orthohyperkeratosis, focal parakeratosis with neutrophils, regular hyperplasia of the epidermis, thin suprapapillary epidermis and dilated blood vessels close to the epidermis and inflammation. Pattern 6 (first biopsy obtained from ID46, HE, original magnification ×40). (**b**): Eczematous-like features with spongiosis and inflammation. Pattern 6 (second biopsy obtained from ID46, HE, original magnification ×18).

Netherton patients carry biallelic mutations in *SPINK5*. All patients (n = 4) with NTS showed a **psoriasis-like pattern** with orthohyperkeratosis, focal parakeratosis and regular moderate hyperplasia of the epidermis (Figure 7) [2]. In one case, neutrophils were found in the SC. One patient had a normal-to-prominent SG. All other patients had at least a partial absence of SG. Spongiosis was present in two cases. Inflammation was moderate in all patients, with one biopsy showing eosinophils. Blood vessels were dilated, showing the kissing phenomenon (n = 4) (vessels close to basal keratinozytes), and the suprapapillary epidermis was thin (n = 3). Follicular hyperkeratosis was present in two patients. The acrosyringium was not assessable.

#### 3.1.6. Peeling Skin Disease

**Figure 8:**
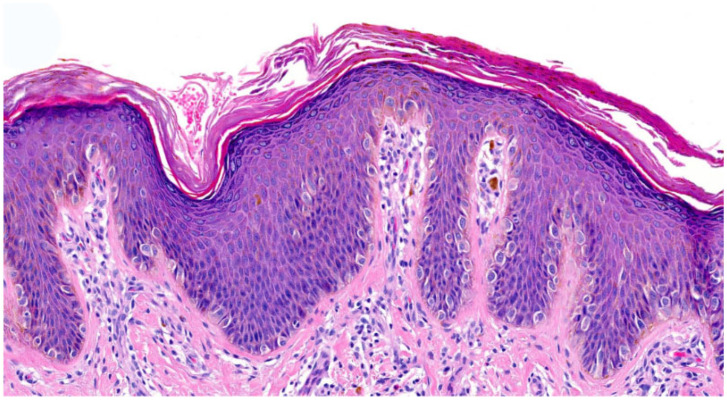
**Peeling skin disease (*CDSN*-nEDD)**. Psoriasis-like pattern similar to Netherton syndrome but with distinct additional features such as subcorneal clefts and acantholytic cells. Pattern 6 (biopsy of a melanocytic nevus obtained from ID50, HE, original magnification ×40).

Peeling skin disease caused by biallelic variants in corneodesmosin shows some clinical overlaps with Netherton syndrome [18]. The patients in our cohort had a **psoriasis-like pattern** similar to Netherton syndrome. Of note, we had 18 biopsies from three patients. Two patients had multiple dark pigmented nevi, which was the reason for multiple biopsies in order to exclude malignancy. The extent of acanthosis varied compared to NTS. We had one biopsy with no acanthosis, six biopsies with mild acanthosis, nine biopsies with moderate acanthosis and two biopsies with severe acanthosis, including eight biopsies with regular hyperplasia and three biopsies with irregular hyperplasia. Subcorneal clefts or acantholytic cells due to corneodesmosin deficiency were prominent in peeling skin disease but were not found in all patients (Figure 8).

#### 3.1.7. Other Syndromic Subtypes

**Figure 9:**
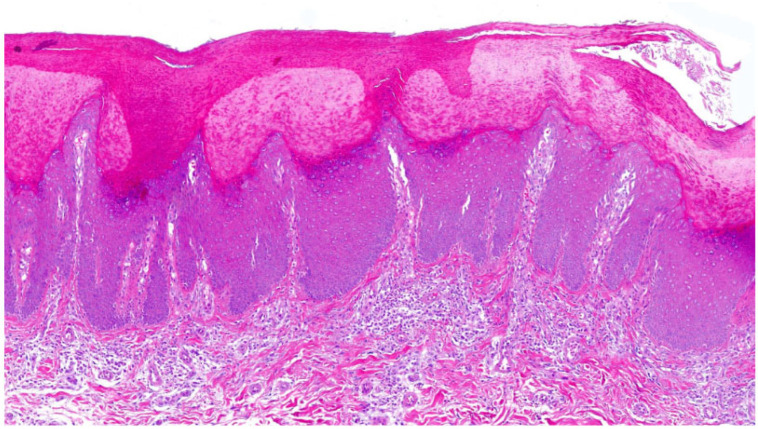
**Congenital hemidysplasia with ichthyosiform nevus and limb defects (CHILD) syndrome (*NSDHL*-sEDD-CHILD)**. Pronounced psoriasiform epidermal hyperplasia with marked orthohyperkeratosis, focal parakeratosis and partial loss of the stratum granulosum. Subepidermal inflammation. Pattern 6 (ID52, HE, original magnification ×15).

Congenital hemidysplasia with ichthyosiform nevus and limb defects (CHILD) syndrome is due to nonsense or missense mutations in the *NSDHL* gene. It is inherited in an X-chromosomal dominant pattern and is therefore usually lethal to males in utero [2]. In our cohort, we found a **psoriasis-like pattern** in our two patients with moderate-to-severe orthohyperkeratosis, focal parakeratosis with neutrophils (n = 1) and a compact SC. The SG was normal or partially absent. Epidermal hyperplasia was moderate and irregular (Figure 9). Blood vessels were dilated with kissing vessels in one case. Of note, xanthoma cells were present in the papillary dermis in only one case.

**Figure 10:**
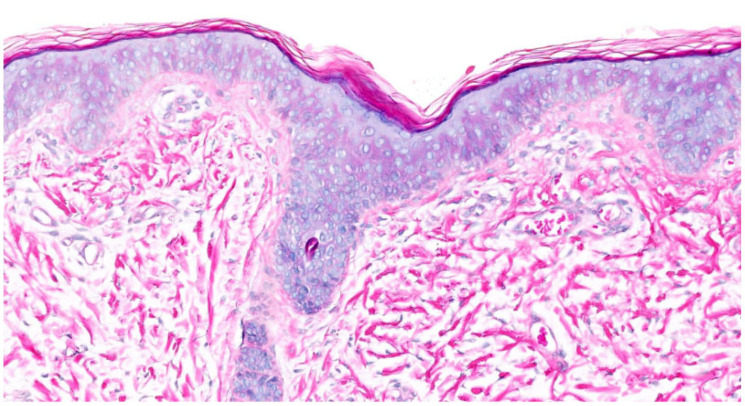
**Ichthyosis follicularis, alopecia and photophobia (IFAP)** (*MBTPS2*-sEDD-IFAP). Orthohyperkeratosis with a basket weave pattern, nearly absent stratum granulosum, follicular hyperkeratosis and rudimentary or atrophic hair follicles. Pattern 1 (ID54, HE, original magnification ×30).

Ichthyosis follicularis, alopecia and photophobia (IFAP) is an X-linked recessive disorder caused by mutations in the *MBTPS2* gene involved in the cholesterol pathway [19]. In our two patients, orthohyperkeratosis was lamellar with a basket weave pattern. The SG was normal in one case and absent in the other patient. Inflammation was absent. The patients had **follicular hyperkeratosis** (Figure 10). In one patient, the hair follicles were rudimentary or atrophic.

**Figure 11:**
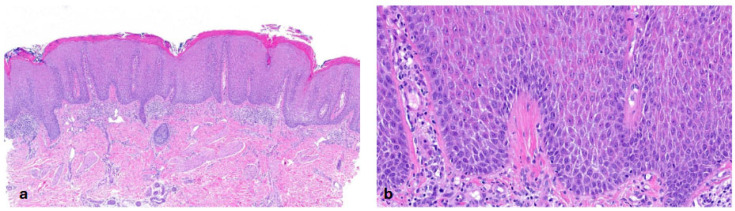
**Severe dermatitis–multiple allergies–metabolic wasting (SAM) syndrome (clinical phenotype) (*DSP*-sEDD)**. (**a**): Orthohyperkeratosis and focal parakeratosis with neutrophilic infiltration, absence of the stratum granulosum, marked regular hyperplasia of the epidermis and characteristic keratinocyte dehiscence associated with moderate inflammatory infiltrate. Pattern 6 (ID56, HE, original magnification ×10). (**b**): High-power magnification reveals suprabasal keratinocyte dehiscence in the absence of additional features of spongiosis. (ID56, HE, original magnification ×40).

Severe dermatitis–multiple allergies–metabolic wasting (SAM) syndrome is caused by mutations in *desmoglein 1* (*DSG1*) or *desmoplakin* (*DSP*). Patients with DSP mutations may have cardiac involvement [20]. Histology shows hyperkeratosis, focal parakeratosis with neutrophils, absent SG, severe regular hyperplasia of the epidermis and typical dehiscence of keratinocytes accompanied by moderate inflammation (Figure 11a,b).

**Figure 12:**
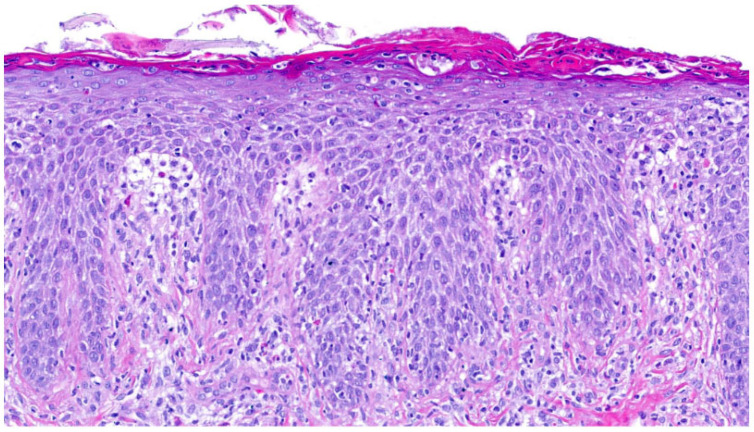
**Mucosa-Associated Lymphoid Tissue Lymphoma Translocation Protein 1 (MALT1) deficiency**. Orthohyperkeratosis with focal parakeratosis and few neutrophils, absence of stratum granulosum and regular epidermal hyperplasia with moderate spongiosis. Blood vessels dilated with severe inflammation. Pattern 6 (ID57, HE, original magnification ×35).

The patient with Mucosa-Associated Lymphoid Tissue Lymphoma Translocation Protein 1 (MALT1) deficiency showed orthohyperkeratosis with focal parakeratosis, few neutrophils, absence of SG and regular epidermal hyperplasia. There were few dyskeratotic keratinocytes and moderate spongiosis. Inflammation was severe with many lymphocytes, erythrocytes and neutrophils, including inflammation of the hair follicles. Blood vessels were dilated without showing a kissing phenomenon (Figure 12).

**Figure 13:**
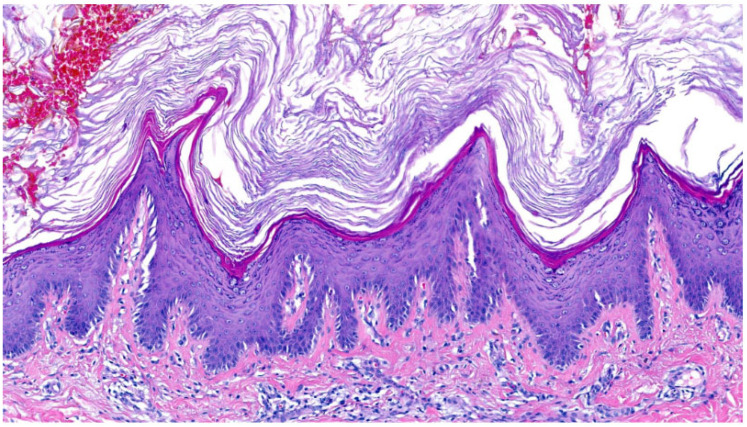
**Keratitis–ichthyosis–deafness (KID) syndrome**. Massive orthohyperkeratosis, mostly lamellar, stratum granulosum prominent, regular acanthosis, papillomatosis with dilated blood vessels and mild inflammation. Pattern 2 (ID58, HE, original magnification ×20).

In keratitis–ichthyosis–deafness (KID) syndrome caused by *connexin 26 (GJB2)* mutations, all patients showed **massive orthohyperkeratosis, mostly lamellar** [2]. SG thickness varied from absent to prominent. Other findings included mild acanthosis, papillomatosis and long and thin rete ridges. Inflammation was mild to moderate. Follicular hyperkeratosis and acrosyringial hyperkeratosis were common. Blood vessels were dilated in two cases. Hyperplastic sweat glands were observed in one patient (Figure 13).

**Figure 14:**
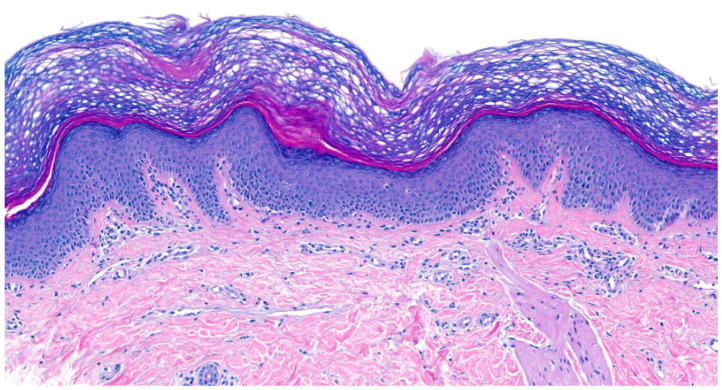
**Sjögren–Larsson syndrome**. Orthohyperkeratosis, stratum granulosum prominent, mild acanthosis, papillomatosis and inflammation. Pattern 2 (ID62, HE, original magnification ×20).

In Sjögren–Larsson syndrome, a neurocutaneous disease due to fatty aldehyde dehydrogenase deficiency (FALDH), **hyperkeratosis with orthokeratosis** was observed [2,21]. The SG was prominent (in one case thinned), and we saw mild acanthosis, papillomatosis (in one patient steeple-shaped appearance) and inflammation (Figure 14). Follicular hyperkeratosis was present.

**Figure 15:**
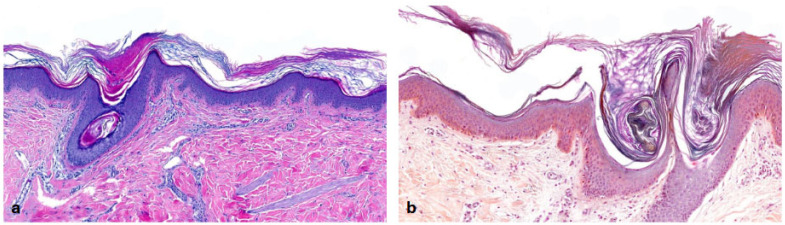
**Conradi–Hünermann–Happle syndrome (*EBP*-sEDD)**. (**a**): Orthohyperkeratosis with a reduced stratum granulosum, follicular hyperkeratosis, mild acanthosis and inflammation. Pattern 1 (ID63, HE, original magnification ×15). (**b**): Calcification of the epidermal and follicular horny layer (ID63, von Kossa stain, original magnification ×30).

Conradi–Hünermann–Happle syndrome is caused by mutations in the *emopamil-binding protein* (*EBP*) gene [2,22]. In our patient, we observed orthohyperkeratosis with a reduced or absent SG. Acanthosis was mild to moderate (Figure 15a). Inflammation was mild, and FHK and AHK were present in one of two biopsies. Of note, calcification of the horny layer of the epidermis and the hair follicles can be demonstrated by von Kossa staining (Figure 15b) [22].

Our patient with neutral lipid storage disease with ichthyosis (Chanarin–Dorfman syndrome) showed lamellar-to-compact orthohyperkeratosis with focal parakeratosis and a normal SG [2]. Acanthosis and inflammation were mild. Basal keratinozytes were slightly vacuolated.

A patient with suspected keratitis–ichthyosis–deafness (KIDAR) syndrome, characterized by deafness, ichthyosis and erythroderma, showed mild orthohyperkeratosis, a prominent SG, mild acanthosis and mild inflammation [23]. Follicular hyperkeratosis and acrosyringial hyperkeratosis were present.

## 4. Discussion

### 4.1. Definition of Six Histological Patterns Including an Algorithm for the Diagnosis of Ichthyosis

Ichthyoses are a very heterogeneous group of cornification disorders with high variability in clinical presentation. There are over 36 entities [4]. Our histological study of 66 patients with well-defined ichthyoses identified six histological patterns that may help to narrow the differential diagnosis (Table 1).

The first pattern is defined by “**orthohyperkeratosis with a reduced or absent stratum granulosum**”, which occurred in IV, XLI, Conradi–Hünermann–Happle syndrome, Sjögren–Larsson syndrome, IFAP syndrome, harlequin ichthyosis, KID syndrome and acquired ichthyosis-like condition (Figure 1, Figure 2, Figure 4, Figure 10 and Figure 15). The latter was not part of our cohort. Acquired ichthyosis-like conditions typically manifest in adulthood and may represent paraneoplastic manifestations or be due to, for example, extreme diets and drug interactions. Some cases are associated with an underlying disease such as venous insufficiency of the leg and mycosis fungoides [24].

Interestingly, the stratum granulosum findings in our sample did not correlate with the number of *FLG* mutations. Therefore, additional immunohistochemistry may be useful in the histology of ichthyosis vulgaris. Keratosis follicularis as a clinical finding may be present in histology and, like palmoplantar hyperlinearity, may be a clinical diagnostic clue. In addition, FLG deficiency in IV can be associated with moderate alterations in epidermal barrier function [25]. Therefore, spongiosis and acanthosis may be present. As follicular hyperkeratosis was common in our overall cohort, clinical features and supportive methods may help to further differentiate between ichthyosis subtypes with follicular hyperkeratosis belonging to the same pattern (Table 3). Notably, inflammatory cells were frequently observed in these non-inflammatory subtypes. Regarding the differentiation between XLI and IV, a reduced SG does not necessarily seem to be a diagnostic clue for IV. Even in some cases with a reduced SG in XLI, we could not find an additional *FLG* mutation implying modifying genetic factors. As an acquired ichthyosis-like condition may also present with massive orthohyperkeratosis with absent or thin stratum granulosum, it cannot be distinguished histologically from inherited forms of ichthyoses (personal observation, DM).

The second pattern is classified by “**orthohyperkeratosis and a well-developed stratum granulosum**”. Our cohort represented a very heterogeneous group of ARCIs, including bathing suit ichthyosis, harlequin ichthyosis, XLI, Sjögren–Larsson syndrome, IFAP, KID, Chanarin–Dorfman syndrome and KIDAR syndrome (Figure 2, Figure 3, Figure 4, Figure 13 and Figure 14). Of note, inflammatory conditions such as lichen simplex chronicus share similarities in the epidermal pattern but differ in the fibrosis of the papillary dermis. Clinical and histological differentiation between XLI and ARCI can be difficult. Clinical features, associated symptoms and electron microscopy may be helpful (Table 2). XLI shows a retention hyperkeratosis which is characterized by the persistence of corneodesmosomes in electron microscopy. Of note, ARCI often showed dilated vessels and the kissing phenomenon even in patients without erythroderma. This correlation should be investigated separately in a larger cohort of ARCI.

**Table 2:** Assignment of six different histological patterns.

Pattern	Clinical Feature	Diagnosis
Pattern I Orthohyperkeratosis with a reduced or absent stratum granulosum	Palmoplantar hyperlinearity, atopy	Ichthyosis vulgaris
	Male patient with gray-to-brown coarse lamellar scaling, flexures spared, improvement in summer, no palmoplantar hyperlinearity; maldescensus testis, hyperactivity, autism, protracted birth; with/without atopy	X-linked ichthyosis
	Late onset of ichthyosis, comorbidities	Acquired ichthyosis
		Conradi–Hünermann–Happle syndrome
	Erythroderma, ectropium, eclabium	Harlequin ichthyosis
		Ichthyosis follicularis with alopecia (atrichia) and photophobia (IFAP) syndrome
	Brownish hyperkeratosis	Keratitis ichthyosis deafness (KID) syndrome
	Cobblestone hyperkeratosis, severe pruritus	Sjögren–Larsson Syndrome
Pattern II Orthohyperkeratosis and a well-developed stratum granulosum	Fine scaling, mild erythroderma	Autosomal recessive congenital ichthyosis (erythrodermic)
	Coarse dark and brown scaling	Autosomal recessive congenital ichthyosis (non-erythrodermic)
	Coarse dark and brown scaling in warmer areas of the body	Bathing suit ichthyosis
	Erythroderma, ectropium, eclabium	Harlequin ichthyosis
	Male patient with gray-to-brown coarse lamellar scaling, flexures spared, improvement in summer, no palmoplantar hyperlinearity; maldescensus testis, hyperactivity, autism, protracted birth; with/without atopy	X-linked ichthyosis
	Cobblestone hyperkeratosis, severe pruritus	Sjögren–Larsson syndrome
		Ichthyosis follicularis with alopecia (atrichia) and photophobia (IFAP) syndrome
	Brownish hyperkeratosis	Keratitis ichthyosis deafness (KID) syndrome
		Chanarin–Dorfman syndrome
		Autosomal recessive keratitis–ichthyosis–deafness (KIDAR) syndrome
Pattern III Hyperkeratosis with ortho- and parakeratosis with preserved or prominent stratum granulosum	Scaly erythema, palmoplantar keratoderma, no systemic manifestation	Erythrokeratoderma variabilis (EKV)
		Keratitis ichthyosis deafness (KID) syndrome
	Dark, coarse scales, lichenification of the dorsum of the hands/feet, knees and plantar hyperkeratosis	Autosomal dominant lamellar ichthyosis
Pattern IV Epidermolytic hyperkeratosis	With palmoplantar keratoderma	Epidermolytic ichthyosis with *KRT1* mutation
	Without palmoplantar keratoderma	Epidermolytic ichthyosis with *KRT10* mutation
		Superficial epidermolytic ichthyosis
		Annular epidermolytic ichthyosis
Pattern V Perinuclear vacuoles and binucleated keratinocytes	Ichthyosis with confetti-like normal skin	Congenital reticular ichthyosiform erythroderma (CRIE)
	Spiny hyperkeratosis	Ichthyosis hystrix of Curth–Macklin
Pattern VI Psoriasis-like features	Ichthyosiform erythroderma, trichorrhexis invaginata, predisposition to infections, food allergies, hypereosinophilia, elevated IgE, failure to thrive	Netherton syndrome
	Erythema, peeling of the skin, allergies, pruritus, hypereosinophilia, elevated IgE, normal body size and weight	Peeling skin disease
		Congenital hemidyplasia with ichthyosiform nevus and limb defects (CHILD) syndrome
	Ichthyosiform erythroderma, alopecia, allergic symptoms, hypereosinophilia, elevated IgE, failure to thrive	Severe dermatitis, multiple allergies, metabolic wasting (SAM) syndrome
	Predisposition to infections, failure to thrive, periodontal disease, enteropathy	Malt 1 deficiency

For the names according to new classification we refer to our manuscript (Materials and Methods, Results) and refs. [1,2].

The third pattern, “**hyperkeratosis with ortho- and parakeratosis with preserved or prominent stratum granulosum**”, was seen in KID syndrome (personal observation, DM) and autosomal dominant lamellar ichthyosis (Figure 5). However, inflammatory skin diseases in general may show a similar pattern, e.g., lichen simplex chronicus and pityriasis rubra pilaris.

KID syndrome shares some features with erythrokeratodermia (EKV): pseudoepitheliomatous, verruciform or psoriasiform hyperplasia with dyskeratosis and rarely perinuclear vacuoles (“bird’s eye”) [9,26]. The orthohyperkeratotic horny layers show focal parakeratosis with focal small roundish nuclear remnants (“shadow nuclei”). Keratotic plugging of hair follicle openings and atrophic sweat ducts are typical. In areas of alopecia, hair follicles are absent or atrophic. Eccrine sweat glands may be reduced in number and/or be atrophic. In general, connexin mutations result in a broad spectrum of histological changes, making a specific diagnosis difficult. Immunohistochemical staining for the expression pattern of connexins may be helpful [26].

The fourth pattern is characterized by “**epidermolytic hyperkeratosis**” (EHK), which occurs in KPI due to mutations in *KRT1*, *KRT10* or *KRT2*, as well as in epidermal nevi, acanthomas, leukoplakia or epidermolytic palmoplantar keratoses [27]. In our cohort, three out of five patients with KPI had non-continuous and suprabasal EHK (Figure 6). Two patients had non-continuous or almost continuous EHK. We could not deduce a specific mutation status from the histological findings, as in superficial epidermolytic ichthyosis, a mutation in *KRT2* may also lead to signs of EHK in the lower parts of the epidermis (personal observation, DM). The patient with clinical findings of superficial epidermolytic ichthyosis carried a *KRT10* mutation. The EHK pattern can also be seen in epidermal nevi, epidermolytic acanthoma, and incidentally in actinic damaged skin, actinic keratosis, invasive squamous cell carcinoma, basal cell carcinoma, epidermal and pilar cysts and various inflammatory diseases [28,29].

Notably, one patient with EI (ID 41) did not show EHK. As this patient had only a mild-to-moderate phenotype, we can speculate that the biopsy was taken from a mildly affected body site.

Annular epidermolytic ichthyosis (*KRT10*-nEDD-annular) is caused by a dinucleotide mutation in *KRT10* and presents with polycyclic, hyperkeratotic plaques predominantly on the trunk and the proximal extremities [28]. With its histology showing epidermolytic hyperkeratosis, it belongs to the group of keratinopathic ichthyoses [28,29]. There are also annular forms with *KRT1* mutations [1].

**Figure 16:**
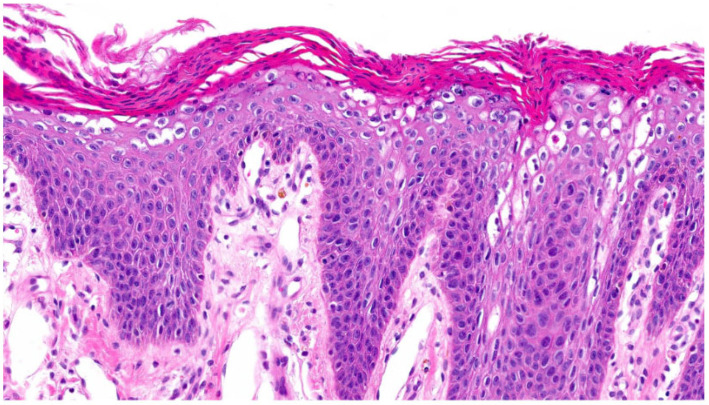
**Congenital reticular ichthyosiform erythroderma (CRIE)**. Hyperkeratosis with large parakeratotic corneocytes, absence of stratum granulosum, regular hyperplasia of the epidermis and vacuolization of the suprabasal keratinocytes, some of which are binucleated. Pattern 5 (HE, original magnification ×40).

The fifth pattern with “**perinuclear vacuoles and binucleated keratinocytes**” is seen in congenital reticular ichthyosiform erythroderma (CRIE) and ichthyosis hystrix of Curth–Macklin (*KRT1*-nEDD-spiny) [29,30]. *KRT10* mutations in CRIE result in almost complete loss of keratin filaments in the perinuclear cytoplasm and vacuolization of suprabasal keratinocytes, some of which are binucleated without eosinophilic intracytoplasmic inclusions. The SG is almost absent, and parakeratotic corneocytes manifest with large nuclei representing transitional cells (Figure 16, unpublished case). Patients with CRIE show spots of normal skin that increase in size over time and appear to be surrounded by erythrokeratotic and hyperpigmented areas in a reticular pattern (“confetti”) during childhood. Notably, the spots of normal skin do not show any pathological changes. Hyperpigmentation of keratinocytes is possible, as is a perivascular lymphocytic infiltrate with melanophages. Histologically, pagetoid dyskeratosis is an important differential diagnosis, defined as an incidental histological finding of no clinical relevance due to friction and maceration with pyknotic nuclei, clear halo, rim of stippled cytoplasm and eosinophilic granules [27,29]. In contrast, ichthyosis hystrix of Curth–Macklin has a *KRT1* mutation in the tail domain that disrupts supramolecular keratin organization, resulting in a shell-like structure of loosely arranged tonofilaments, but unlike CRIE, it forms an SG and an orthohyperkeratotic horny layer [27,30].

The sixth pattern is characterized by “**psoriasis-like features**”, which in some cases are indistinguishable from psoriasis vulgaris (Figure 7, Figure 8, Figure 9, Figure 11 and Figure 12) [31]. This pattern contributes to an entirely new understanding of the overlap between different inflammatory skin conditions. The histology of Netherton syndrome (NS) can be similar to that of psoriasis vulgaris, except NS has a very thin stratum corneum in early life. However, clinical aspects (Table 2) are not usually characteristic of psoriasis and provide additional clues [31]. Interestingly, the histological spectrum of NS is broad and includes atopic dermatitis-like features with spongiosis (Figure 7) [8,31]. Immunostaining for LEKTI allows a reliable specific diagnosis of NS by showing loss of LEKTI in the epidermis and hair follicle epithelia [8]. Other psoriasiform dermatoses may exhibit reduced LEKTI expression in the epidermis, while hair follicles remain distinctly immunoreactive.

Peeling skin disease (PSD) is characterized by lifelong patchy erythema with peeling of the skin, as with NS patients, who are at high risk of food allergy, pruritus and asthma. Histologically, the subcorneal clefts in psoriasiform dermatitis have been difficult to recognize (Figure 8). Sometimes step sections are helpful. We observed erythrocytes in the subcorneal clefts, derived from the biopsy procedure, which may serve as a diagnostic clue to exclude artificial clefting after tissue processing. Immunohistochemical staining for corneodesmosin may also help to differentiate PSD from other ichthyoses.

In CHILD syndrome, xanthomatous cells in the papillary dermis, if present, are a helpful histological clue when present in association with a psoriasiform pattern (Figure 9) [32]. In addition, immunostaining for adipophilin can highlight lipid inclusions in fibroblasts.

Severe dermatitis–allergies–metabolic wasting (SAM) syndrome presents with congenital erythroderma reminiscent of Netherton syndrome. Food allergies, recurrent skin and respiratory infections, failure to thrive and growth retardation may cause severe problems. Skin biopsy shows psoriasiform dermatitis with additional histological evidence. Mutations in desmosomal proteins can lead to the histological finding of “desmosomal-type acantholysis”, defined by hypereosinophilic epidermal keratinocytes, widening of intercellular spaces and partial loss of intercellular bridges in the suprabasal layers (Figure 11) [33]. Immunostaining for desmoglein or desmoplakin may help to rapidly establish the diagnosis (personal observation).

MALT1 deficiency also presents with postpartum periodic erythroderma with scaling and recurrent systemic infections and severe internal complications. Histology is characterized by psoriasiform and spongioform dermatitis, which can be easily differentiated from NS by immunohistochemistry for LEKTI (Figure 12).

As a result of our cohort analysis, we suggest that **follicular hyperkeratosis** not be defined as an independent pattern, as it may be associated with all subtypes of ichthyosis (Table 1 and Table 2). Furthermore, pachyonychia congenita and ectodermal dysplasia may show aspects of follicular hyperkeratosis (Table 3). **Hyperkeratosis of the acrosyringeum** is also a variable feature in many ichthyoses and is thought to lead to impaired sweating. However, we were not able to systematically analyze this criterion, as the sections did not show a significant number of sweat gland orifices.

**Table 3:** Differential diagnoses of follicular hyperkeratosis.

Follicular Hyperkeratosis in Ichthyoses
Ichthyosis vulgaris with follicular keratosis
AR-congenital ichthyosis
Sjögren–Larsson syndrome
Ichthyosis follicularis with alopecia and photophobia
Hystrix-like ichthyosis with deafness (HID) and keratitis ichthyosis deafness (KID) syndromes
Other ichthyoses (see Table 1)
**Follicular hyperkeratosis in other hereditary cornification disorders**
Pachyonychia congenita Morbus Darier Warty dyskeratoma Keratosis lichenoides chronica Keratosis pilaris atrophicans Bazex–Dupré syndrome Congenital alopecias Hereditary mucoepithelial dysplasia Porokeratosis Porokeratotic adnexal ostial nevus (PAON) Naevus comedonicus Familial dyskeratotic comedones

In summary, the value of histological patterns in diagnosing inherited ichthyoses is underestimated. Here, we describe a practically relevant algorithm consisting of six histological patterns. In settings lacking access to genotyping, the analysis of skin biopsy sections by light microscopy can aid in narrowing down the differential diagnosis. If available, molecular findings and other laboratory values combined with clinical clues and histological examination not only support the diagnostic process but also increase our understanding of the consequences of the genotype for the phenotype (ranging from molecule-level effects to tissue morphology).

### 4.2. Limitations

We could not include all of the diagnoses listed in the consensus nomenclatures for epidermal differentiation disorders, previously referred to as ichthyotic skin diseases [1,2,6]. This was due, among other things, to the lack of histological examination in confirmed cases or the lack of genetic results and other diagnostic methods. Therefore, we deliberately did not include erythrokeratodermia (EKV). Recently, an EKV-like phenotype has also been shown in *kallikrein (KLK)11* mutations, which were initially not yet part of the panel diagnosis [34]. For the sake of completeness, we have included some diagnoses that were missing in our cohort in our diagnostic algorithm. The criteria for these diagnoses are based on experience with consultation cases of ichthyosis and findings in the literature that provide convincing histological descriptions.

Moreover, due to the rare-to-ultrarare nature of the diseases investigated, the number of available patients is limited, and subtype representation may be uneven. Nevertheless, the overall number of patients and the inclusion of a well-characterized cohort from specialized reference settings provides meaningful insights into conditions for which comprehensive datasets are otherwise scarce.

Notably, some diagnoses present with variable patterns, making differential diagnosis difficult. As we usually only look for distinct mutations in genetic analysis, we cannot completely exclude that patients have other modifying genetic factors outside of the known genes for genodermatoses. Further studies correlating the phenotype, mutational status and histological patterns in larger cohorts of patients are needed to understand the clinical and histological heterogeneity of cornification disorders. The importance of immunological mechanisms and modifier genes, as well as environmental factors, contributes to this heterogeneity. Recently, a highly variable response to biologic agents has been demonstrated, underscoring this heterogeneity [35]. In addition, variations in histology may be related to the biopsy site, age of the lesion or pretreatment.

## 5. Conclusions

In this study, we propose histological patterns and diagnostic pathways that may help to improve the diagnosis of ichthyoses, some of which are rare, syndromic and life-threatening.

## Data Availability

All data from this study is provided within the manuscript.
